# Insights from social media into public perspectives on investigative genetic genealogy

**DOI:** 10.3389/fgene.2024.1482831

**Published:** 2025-01-06

**Authors:** Sara Huston, Diana Madden, Andrea Villanes, Nathan Reed, Whitney Bash Brooks, Christopher Healey, Christi Guerrini

**Affiliations:** ^1^ Mary Ann & J. Milburn Smith Child Health Outcomes, Research, and Evaluation Center, Ann & Robert H. Lurie Children’s Hospital of Chicago, Chicago, IL, United States; ^2^ Department of Pediatrics, Northwestern University, Chicago, IL, United States; ^3^ Department of Computer Science, Institute for Advanced Analytics, North Carolina State University, Raleigh, NC, United States; ^4^ Baylor College of Medicine, Center for Medical Ethics and Health Policy, Houston, TX, United States

**Keywords:** public understanding of science, social media, kinship analysis, DNA testing, forensic DNA, investigative genetic genealogy

## Abstract

Social media sites like X (formerly Twitter) increasingly serve as spaces for the public to discuss controversial topics. Social media can spark extreme viewpoints and spread biased or inaccurate information while simultaneously allowing for debate around policy-relevant topics. The arrest of Joseph J. DeAngelo in April 2018 ignited a barrage of social media conversations on how DNA and genetic genealogy led to the suspect. These conversations continued over the following years as policies changed and as the use of the approach expanded. We examined social media coverage of investigative genetic genealogy (IGG) to characterize the volume and temporal patterns in the topics and sentiments of these public conversations. First, using a data analytics tool Brandwatch Consumer Research, we built flexible search strings to collect tweets from the social media platform Twitter/X for IGG-relevant content published from 2018 to 2022, resulting in 24,209 tweets. Second, we applied informatics tools to the dataset to generate topic clusters and analyze trends in cluster volume and distribution over time to define the top 25 peaks in tweet volume, representing the 25 events that generated the highest volume of conversation over the 5-year period. Third, drawing on the contextual framework of key IGG events, we selected three of the top ten events to code for sentiment along with a randomly sampled subset of tweets across the timeframe. Qualitative coding for position on IGG revealed a majority of tweets were supportive of the use of IGG, but key concerns were also voiced about the ethics of IGG. Over a third of conversations on Twitter/X were on either cases solved or suggestions for use of IGG. We archived the social media data for future research. These data highlight key areas of public support and concern within IGG processes and across application contexts.

## 1 Introduction

In 2018, the arrest of the Golden State Killer made headlines around the world. Joseph James DeAngelo, who eventually confessed to committing 13 murders and over 45 rapes, was identified using an approach called investigative genetic genealogy (IGG) (Fieldstadt, 2020). This approach involves uploading data from crime scene DNA to genetic genealogy databases with the intention of identifying a criminal offender’s genetic relatives and, eventually, locating the offender in their family tree (Greytak et al., 2019). Since DeAngelo’s arrest, use of IGG has spread across the United States, aiding resolution of hundreds of various types of cases (Dowdeswell, 2023; Katsanis, 2020). At the same time, its ethical and legal appropriateness is being challenged by arguments that it violates fundamental privacy interests of database participants and their families (Brown, 2019; Fullerton and Rohlfs, 2018; Berkman et al., 2018; Murphy, 2018). The privacy concerns are exacerbated by the commercial success of the genetic genealogy industry; nearly 50 million DNA profiles have been created, with the largest database containing over 25 million DNA profiles (ISOGG, 2024; Larkin, 2021). Researchers have projected that 60% of individuals of European descent have relatives in a database comprised of DNA data from only 1.28 million individuals (Erlich et al., 2018).

ELSI studies have found that the public is generally concerned about genetic privacy but many are willing to share their genetic data for purposes they consider worthy. Notably, none of those studies asked about use of genetic data in IGG (Oliver et al., 2012; Robinson et al., 2016; Skinner et al., 2008; Nagaraj et al., 2014). Most of these studies pre-date IGG and none asked about use of genetic genealogy databases by law enforcement. In 2018, Guerrini et al. conducted the first survey to assess public opinion of IGG and found strong support when it is used to identify perpetrators of violent crimes (Guerrini et al., 2018). However, the survey was administered shortly after DeAngelo was arrested, when the privacy implications of IGG were just beginning to be understood by experts and the public, and it was not designed to account for the complex trade-offs that the public makes when assessing the value and acceptability of any use of genetic data. Given that IGG could implicate most of the U.S. population in the near future, it is critical to address public concerns and develop policies and practices that are informed by a broad base of evidence on public perspectives. Ultimately, any policies and practices must balance safety and privacy in a way that is acceptable to the public.

Social media platforms are a unique source of this information and have the advantage of facilitating observations of conversations as they emerge, without researcher intervention or influence (Newman, 2017; Ronzhyn et al., 2023). These data allow for the study of public attitudes over time, including identification of trends. This can be especially useful in investigations of the ethical, legal, and social implications (ELSI) of genetic technologies: perspectives on technologies can change as the public becomes more familiar with them and learns about new applications. Understanding public sentiment over time is particularly informative for policy considerations because it captures these shifts (Liu, 2015).

Just like any other messaging, journalistic decisions about how IGG is framed in the headlines and in the body of articles or broadcasts affect public opinion (Entman, 1993). The term “framing” describes how journalists intentionally situate information in a narrative context; framing helps the reader absorb and interpret information, but it also imprints it with a message (Entman, 1993; Price, Tewksbury, and Powers, 1997; Edgerly et al., 2013). That message includes an emotional context to elicit sentiment (Liu, 2015), which is useful for conveying facts that are difficult to follow since readers react best to a narrative (Simis-Wilkinson et al., 2018). This journalistic packaging is especially important with respect to DNA testing-related news as some DNA testing practices carry connotations that can fuel controversy; the potential for controversy elevates the role of framing in shaping public approval and tolerance of potentially sensitive or intrusive DNA uses (Condit et al., 2001; Nelson et al., 1997). Social media amplifies news media, serving as a platform for spreading information and discussing or debating content (Thorson et al., 2013; Shapiro and Hemphill, 2017). Often a social media post is the headline of a news article, with or without the link to that article. The mass distribution of news enabled by social media provides new opportunities for members of the public to re-frame messages originally framed by journalists (Bruns, 2018; Thorson et al., 2016). Both journalistic framing of events and users’ exchanges can shape public perception and even policy as individuals, organizations, and governments interact with social media.

The number of social media posts in social media spike following media coverage of new applications of DNA technologies. Our team observed anecdotally how conversations around IGG surged with new media coverage over a period of years starting on 25 April 2018 with the arrest of the Golden State Killer. We suspected that misinformation could spread if or when news coverage failed to provide accurate information on IGG and that chronicling these conversations could help to understand the sources and scale of misinformation.

We partnered data analytics methodology with traditional qualitative social science research (Madden et al., 2024) to capture social media content on IGG conversations over the first 4+ years of its use by law enforcement, archived the content for future study, and characterized the topics and sentiment of those conversations (Andreotta et al., 2019). Our interest was to examine conversations in social media that have been ongoing as practice and policy develop and to start to characterize those conversations. We characterized four dimensions of conversations on IGG: (1) topics and subtopics related to use of IGG; (2) purpose of tweets on IGG; (3) sentiment or emotional tone of tweets; and (4) position or attitude toward IGG. The data archived from our searches could be mined for additional information in the future, such as topic-specific analysis including research into misconceptions and misinformation; the data were made publicly available to enable further research (https://osf.io/n9tfu/).

## 2 Methods

We conducted data collection and analysis in four stages: (1) selection of platforms; (2) search string development; (3) topic clustering and analyses; and (4) sentiment analyses. We use the following terms to describe the data: “**social media post**” is used as a general term for a message on any social media platform; “**tweet**” refers to the short message by a single user on Twitter/X; “**post**” refers to any tweet that is original content on Twitter/X; “**reply**” refers to a tweet on Twitter/X that is posted in response to another tweet; “**share**” or “**re-tweet**” refers to a tweet that is re-posted; “**peaks**” refers to the temporal spikes in conversations captured on Twitter/X; “**bins**” refers to the temporal clusters of data to break up the analyses; “**codes**” refers to the label or category assigned to a piece of qualitative data.

### 2.1 Social media platform selection

There are several popular social media platforms available, so to select the appropriate platforms for IGG analyses, we considered four domains. First, we considered how accessible the data were, as many social media platforms keep data behind paywalls. Second, we were interested in the platforms to which users might turn for news, information, and policy discussion. Third, we focused on text-based user activity as opposed to content that is primarily photographs or videos. Fourth, we wanted to select a platform where users were discussing IGG.

In the 6 years since the arrest of DeAngelo, practice and policy have been rapidly developing, partly fueled by public fears (Guerrini et al., 2021). We selected Twitter/X (San Francisco, CA) for our focus because the platform facilitated contemporary commentary and consumption of news about IGG in the timeframe of our study. Twitter also has historically been a site where activists, politicians, and governments have been engaged (Shapiro and Hemphill, 2017; Puschmann et al., 2013).

We wanted to be able to search and download tweets including historical data and to access the full text of tweets and associated metadata (e.g., how many people interacted with the tweet, the geographical location where it was tweeted). This level of data access is not free to the public. Through collaborators, we were able to use the subscription-only social media software platform Brandwatch Consumer Research (Brandwatch.com, Brighton, United Kingdom) to search and collect tweets. Brandwatch has agreements with various social media companies to provide data access *via* its proprietary tools for search and analysis; it commonly is used for marketing analysis to understand consumer interests. In October 2022 when our search was conducted, Brandwatch provided access to Twitter/X, among other social media sites (e.g., Reddit), and a subset of blogs, news headlines, and other forums. Brandwatch did not grant access to Facebook, but at the time of the search, it did include full historic data and full text access for Twitter/X, along with metadata (e.g., usernames, location). We note that we selected Twitter/X as a platform and searched and collected data prior to the acquisition of Twitter by Elon Musk on 28 October 2022 (Siddiqui and Dwoskin, 2022), and before the transition of the company name from Twitter to X Corp in July 2023. These transitions were accompanied by a marked shift in policies that affected the userbase and changed data access (Dang, 2023). Future researchers will want to assess social media sites at the time of research.

Permissions for access to Facebook (*via* Meta, Cambridge, MA) are more restrictive than Reddit and Twitter/X in that individual contributors must provide permission to access their data. We considered capturing publicly available Facebook data; however, being aware that the IGG-related conversations on the platform take place largely in private Facebook groups, we reasoned that the publicly available data would not be a rich source of public conversation. We also considered adjusting our exempt human subjects protocol, which prohibits direct contact with social media users, to allow us to request research access to IGG-related community Facebook administrators but reasoned that we were unlikely to gain access to all relevant groups and thus would have an incomplete dataset. Since our goal was to capture an unbiased and comprehensive dataset from our chosen social media platforms, including Facebook was incompatible because of these restrictions. A future project might include digital ethnography on IGG-related communities of interest, especially with data captured from communities on Reddit and Facebook, but for our purposes, we focused on Twitter/X.

### 2.2 Twitter/X search string development

We crafted a single search string for Twitter/X to return as much relevant content as possible while minimizing irrelevant content containing coinciding terms. Using a single search to capture the full dataset, as opposed to multiple searches, limited the possibility of duplicate tweets in the dataset, creating the most relevant, comprehensive, and clean dataset possible for analysis and archiving for posterity and future research. We used a systematic approach to develop the search string.

First, we used small-scale test searches on the publicly available Advanced Search interface of Twitter to see if the topic of IGG was being discussed and to become familiar with how it was being discussed. Next, researchers with topical expertise brainstormed potential search terms directly related to IGG and potential pairings among groups of terms (see Supplementary Table S1). We brainstormed 34 terms related to investigative genetic genealogy, law enforcement, DNA, and genealogy (see Supplementary Table S1). Four of the terms were general terms related to IGG; terms in the other three categories (law enforcement, DNA, and genealogy) were combined in various forms to create 73 pairings that targeted our topic of interest.

We then applied a “pre-search” methodology (Nelson et al., 1997) to test the utility of the 34 individual terms and 73 pairings of terms as search string components, again using Twitter’s Advanced Search interface (Snelson, 2016). Filters were set to include tweets that were in English, U.S.-based, and in our date range of interest (1 April 2018 to 25 October 2022). We also used Twitter’s quality filter, which filters duplicate content and content that appears to be automated based on account origin and behavior (Wagner et al., 2019).

To estimate the utility of each term or term pairing, we noted the date of each test search, coded the “latest” (Twitter/X’s term for display of search results descending from most recent) 10 tweets for relevance to IGG and noted the age of the 10th (or oldest) tweet and the length of time between the search date and oldest tweet (see Supplementary Table S2) (Madden et al., 2024). These searches were conducted between 29 August 2022 and 14 September 2022. Of these searches, 35/73 (47.9%) had 10/10 relevant content related to IGG so were included in the final search string; 12/73 (16.4%) had 0/10 relevant content, so were excluded from the final search string. Any searches that yielded fewer than 10 total tweets ever (5/73, 6.8%) were included in the search string. Any remaining searches with fewer than 5/10 relevant tweets (11/73, 15.1%) were excluded from the final search string. The remaining 10 search result sets were scrutinized for overlap in content and excluded if the results were captured in more than one search (5/73, 6.9%). We ultimately identified 44 combinations of terms (44/73, 60.3%) that returned results of sufficient relevance to be included in the search string.

We iteratively refined our pool of terms using what we learned from engaging with data, taking into consideration tweet components such as hashtags and direct mentions as well as commonly misapplied terms. One example of this is that we identified that “familial searching” was often mistakenly used as a proxy for IGG, prompting us to include it in our pool of terms. We also took into consideration common misspellings of relevant terms, such as “Family Tree DNA” versus “FamilyTreeDNA.”

To craft the final search string, we added Boolean operators to appropriate words to be inclusive of alternate forms, identifying the word stems (e.g., “famil*” for “family” and “familial”), and compiled the search string according to Brandwatch requirements (see Figure 2). We set search filters in Brandwatch to replicate our test searches and applied additional filters provided by Brandwatch to exclude common sources of irrelevant tweets, such as advertisements. Our Brandwatch search results were exported and spot-checked to ensure they contained relevant data without duplicate tweets.

#### 2.2.1 Peak analyses

**FIGURE 1:**
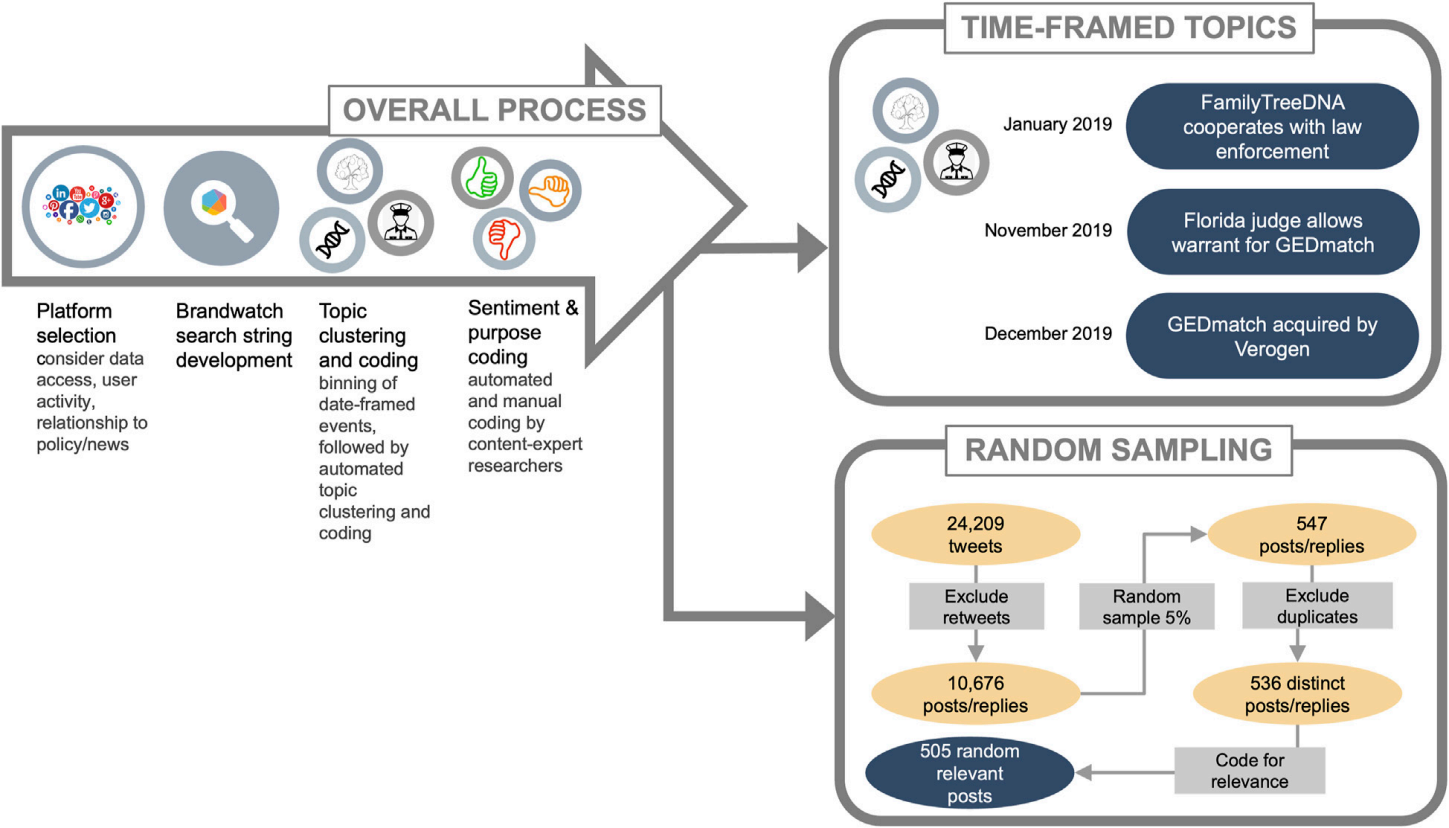
Workflow for capturing and sentiment analyses of IGG conversations on social media. The process started with selection of a platform for analyses, development of a comprehensive search string, topic clustering and coding, then sentiment and purpose coding. These analyses were conducted on two datasets: time-framed topics with significant number of tweets and a random sample set across the timeline.

Our full dataset numbered in the tens of thousands of tweets, precluding a purely manual approach to identifying topics. We applied a combined data analytics and manual review strategy to group topically-related tweets into “topic clusters” (see Figure 1 for schematic) (Madden et al., 2024).

Topic clustering is a data analytics approach that uses an algorithm to group tweets containing similar terms within a large dataset (Madden et al., 2024; Villanes et al., 2018). Because our dataset spanned 4.5 years (55 months), we divided the data into smaller timeframes prior to automated topic clustering; we did this to minimize the potential for tweets with similar language but discussing discrete events to be grouped together (Madden et al., 2024).

The full dataset showed spikes in the volume of tweets over time, which we call “peaks.” We hypothesized that the most pronounced peaks represented discussion of discrete “events” as they appeared in the news cycle. Dividing the data into even time spans across the 55 months would have split the content of some peaks between datasets, potentially resulting in multiple, smaller clusters on similar topics. Instead, we used the start and end dates of each peak to determine where to divide the data. We call these subsets “bins”.

Automated topic clustering was applied to the bins. Over half of the full dataset was made up of shares or “retweets”, and automated topic clustering successfully grouped these tweets together within each bin. Unique tweets with similar wording to the shares or retweets also formed part of these clusters. In some cases, bins contained one cluster significantly larger than the others; to group the remaining tweets into more homogenous clusters, four of the nine bins underwent a second round of topic clustering with the dominant cluster removed.

Coders with expertise in IGG then manually reviewed the contents of the resulting topic clusters (Madden et al., 2024). As we hypothesized, clusters tended to form within the start and end date of the peaks contained within bins. Coders identified the subtopics that made up most of the volume of each peak; we call these time-specific subtopics “events”. The peak analyses strategy for this study is described in Madden et al., 2024; Madden et al., 2024). While the automated topic clustering was successful in creating initial groups of tweets, we expected that tweets pertaining to a peaks’ event but using distinctive wording might not have been included in its cluster. Once manual coders had identified the defining event for each of the peaks, we identified event-related terms to pull additional tweets within each peak’s respective bin as a supplement to the automated topic clustering.

#### 2.2.2 Random sampling

While surges in volume at specific points in time around a single topic were one of the defining characteristics of our data, we also wanted to capture any topics that might not have peaked temporally but emerged over time and to identify shifts in topics over time. To do this, we devised a sampling strategy to randomly sample tweets across the full dataset, which spanned 4.5 years. To avoid retweets that comprised the events identified in the peak analyses, we excluded retweets during sampling.

We then used a random number generator to select among all unique posts and replies across the dataset. We selected tweets until 5% of the posts and replies were represented in the random sample. Thus, the sample included approximately 1 tweet every 3 days and 2–37 tweets per month, depending on the density of conversations. These tweets were manually coded for relevance, topic/subtopic, purpose, and sentiment.

#### 2.2.3 Topic coding

A study team member assigned codes for subtopics to each of the randomly sampled tweets. A second content expert reviewed the subtopic codes and corrected the wording of the subtopic codes as appropriate, then combined subtopics into codes for broad topics. For the tweets in the random sample set that were already coded as one of the peak events, these could be assigned the subtopic code from the event.

### 2.3 Twitter sentiment analysis

#### 2.3.1 Codebooks and rulebooks

Codebooks assisted manual coding of sentiment, purpose, and IGG position. Existing codebooks and rulebooks for code application from prior unpublished research were adapted for sentiment and purpose coding; the IGG position codebook was developed for this project (see Supplemental file for codebooks). The initial codebooks were adapted throughout the coding process to accommodate details not considered by investigators at the outset. All coding was completed by two coders independently and then reconciled through comparison and discussion.

#### 2.3.2 Tweet purpose coding

We adapted a codebook from a prior study to identify how tweets function in social media dialogue, for instance to share news or personal opinions (Madden et al., 2024). Coding for purpose allows us to characterize the composition of a random sampling of tweets. Purpose codes were based on interpretation of the text alone, except for distinguishing professional from personal opinions, which requires review of the Twitter user profile, when it is available (Madden et al., 2024). Two content experts coded for purpose and compared for resolution, using discussion or a third expert coder to resolve any discrepancies.

#### 2.3.3 Tweet sentiment coding

For this study we used both automated sentiment analyses and researcher coding to examine the dataset (Madden et al., 2024). We then could compare the results of the automated analysis to the researcher coding. Our coding methodology prioritizes how social media posts are perceived by an average reader rather than the intentions of the Twitter/X user that authored a tweet. This allows characterization of public discourse without delving into specifics of individual users, who might be people, organizations, or bots.

##### 2.3.3.1 Tweet automated sentiment analysis

Brandwatch’s proprietary automated sentiment analysis model (version October 2022) assigns a sentiment code of positive, negative, or neutral to every tweet based on natural language processing of the text (Soroka et al., 2015).

##### 2.3.3.2 Tweet researcher-coded sentiment analysis

Since the Brandwatch algorithm is not sophisticated enough in its lexicon to reliably code for topic-specific sentiment (Madden et al., 2024), we manually coded the sentiment of our random sample of tweets for comparison to the automated sentiment codes.

##### 2.3.3.3 Tweet researcher-coded IGG position analysis

Because IGG was relatively new to the public and the policies and practices were shifting rapidly within the timeframe of our dataset, we hypothesized that the tone of each tweet might not always reflect the tweet’s perspective on the overall subject area (Madden et al., 2024). For example, a person might express their anger at the way a murder investigation was managed (negative sentiment) but be supportive of the use of IGG in the case (receptive sentiment). We used the term “receptive” to encompass the range of support from acceptance to excitement; similarly, “hesitant” was used to encompass the range of discomfort from mild concern to adamant opposition (Madden et al., 2024). We used the term “impartial” to differentiate the code from “neutral” text sentiment. Two content experts independently coded the data and then compared any discrepancies for resolution, using discussion or a third expert coder. The coding process involved careful examination and interpretation of the content to ensure accurate representation of the underlying themes or concepts. The IGG position coding strategy for this study is described in Madden et al., 2024; Madden et al., 2024).

### 2.4 Quantitative analysis

Microsoft Excel was used to tabulate codes and generate graphical representations of the data. Statistical analysis was conducted using Excel to calculate descriptive statistics, such as frequencies and percentages. To determine the concordance of manual coding to automated coding, the percentage of matching codes was calculated.

### 2.5 Human subjects considerations

This study protocol and the study activities to be performed at Lurie Children’s by its researchers were determined to meet exemption criteria of 45 CFR 46.104(d) (2) by the Lurie Children’s IRB (#2021–4,550).

## 3 Results

### 3.1 Final search string and results

**FIGURE 2:**
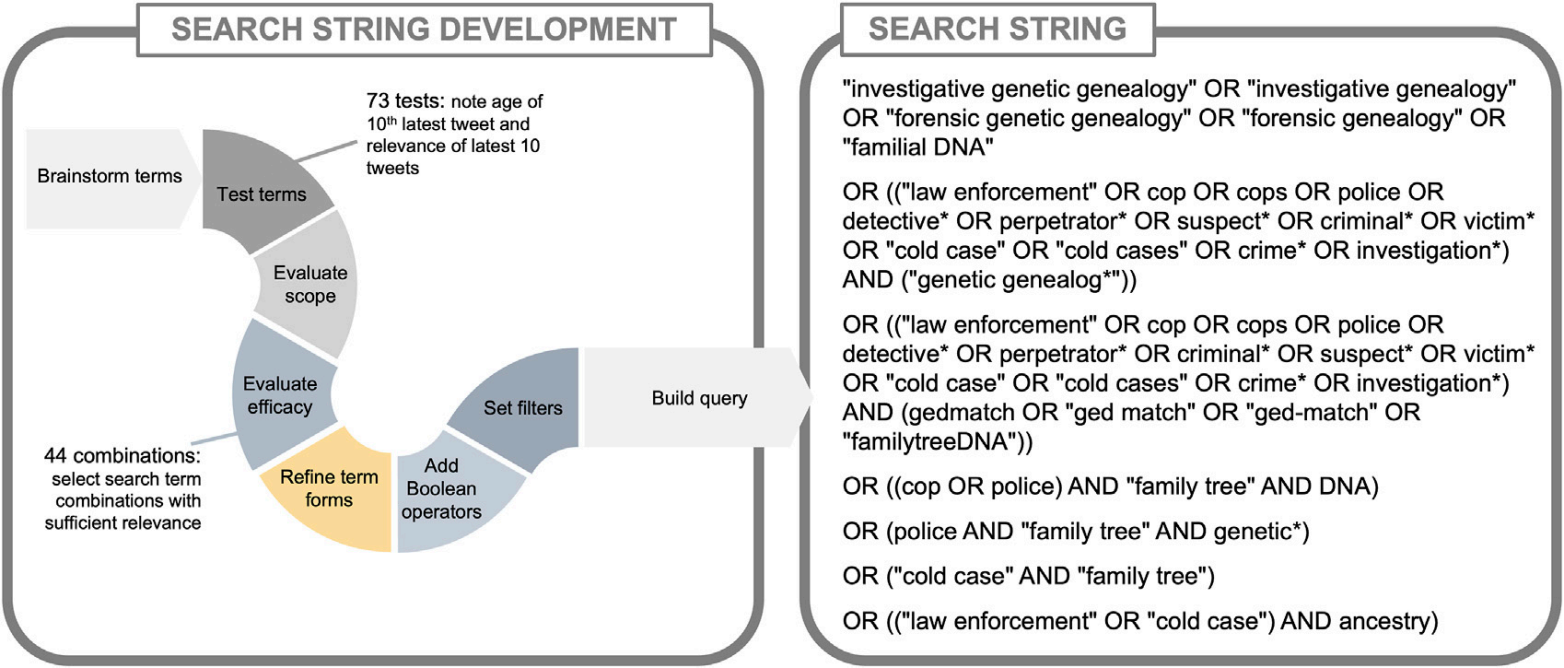
The Brandwatch search string for Twitter/X from 01 April 2018 to 25 October 2022 with filters for English language and U.S.-based.

The finalized search string entered into Brandwatch (see Figure 2) resulted in an Excel format (.cvs) file with 113 columns overall, 16 with types of data specific to Twitter/X. A total of 24,209 tweets were captured; 56% were retweets, 12% replies, and 32% original tweets (Supplementary Table S3); 77.5% of the tweets contained a URL. Authors of the tweets were 15,024 unique users. A slight majority of tweets were denoted by Twitter/X as female (53%) (Supplementary Table S3) and a majority (88%) were denoted as from individuals rather than organizations. According to our topic coding of the random sampling, 94.2% of the collected tweets were relevant to IGG.

### 3.2 Topic analysis

#### 3.2.1 Events and subtopics identified in peak analyses

**FIGURE 3:**
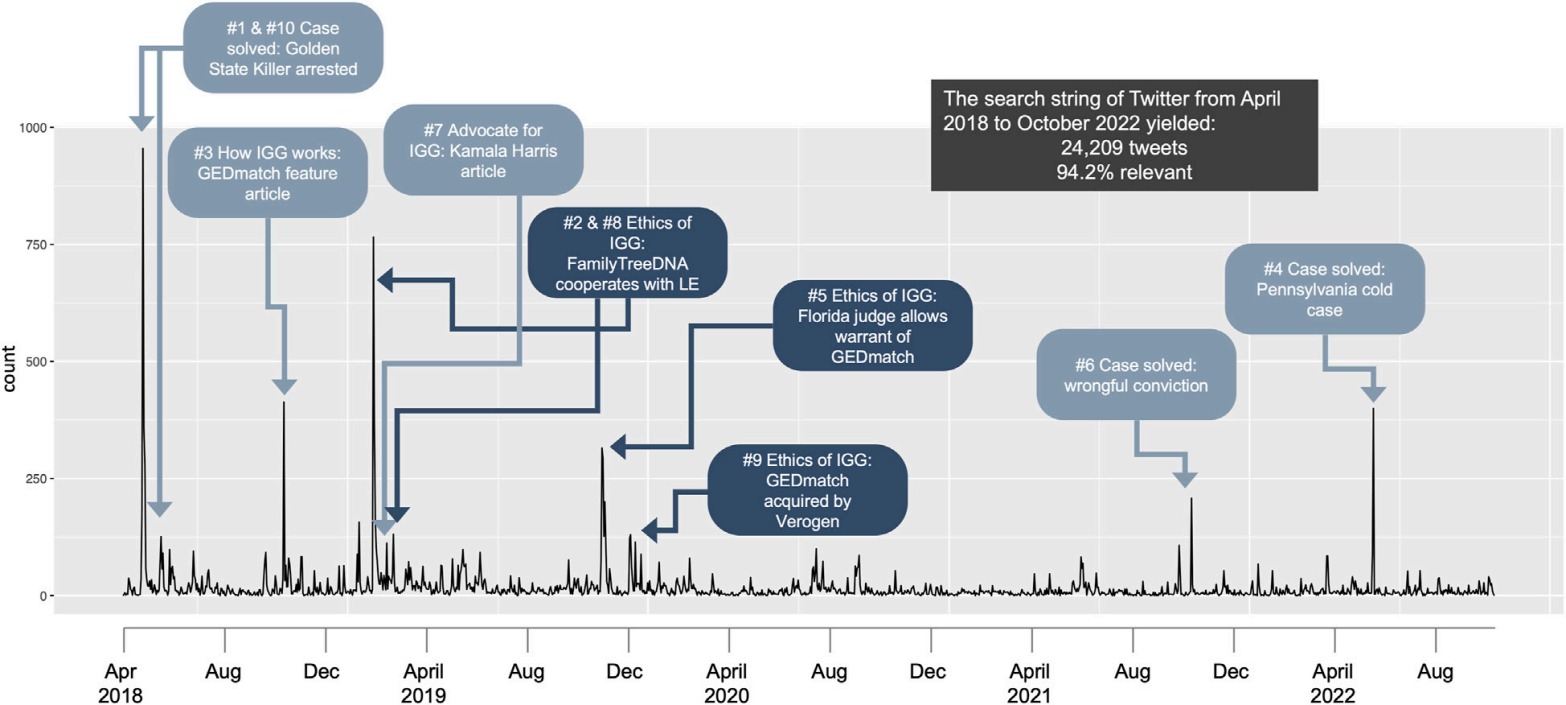
Key temporal events revealed in automated topic clustering.

We identified the 25 highest peaks in the full dataset (see Supplemental Figure S1) and divided the data into 9 timebound subsets of data (Supplementary Table S5), each of 12 months or less with fewer than 6 peaks. The 10th highest peak contained just over 100 tweets, whereas the 11th highest peak was shortly after the arrest of the Golden State Killer with about 100 tweets. We opted to narrow the efforts of manual coders to the top ten peaks, each of which corresponded with an event (see Figure 3). This resulted in a pool of 3,313 tweets total. Using the event terms to search within the bins expanded the 10 clusters from 3,313 tweets to 5,004 tweets. 4,892 (97.8%) of these tweets coded as relevant to the subtopic events. Three of the ten event subtopics were “ethics of IGG” topics, which were selected for further manual analyses since they were conversations on IGG processes that might be relevant to public opinion and policy, These three topics were (1) “FamilyTreeDNA cooperates with law enforcement” (FTDNA-LE), (2) “Florida judge allows warrant for GEDmatch” (FL warrant), and (3) “GEDmatch acquired by Verogen” (GEDmatch acquire).

#### 3.2.2 Subtopics identified in random sampling

**FIGURE 4:**
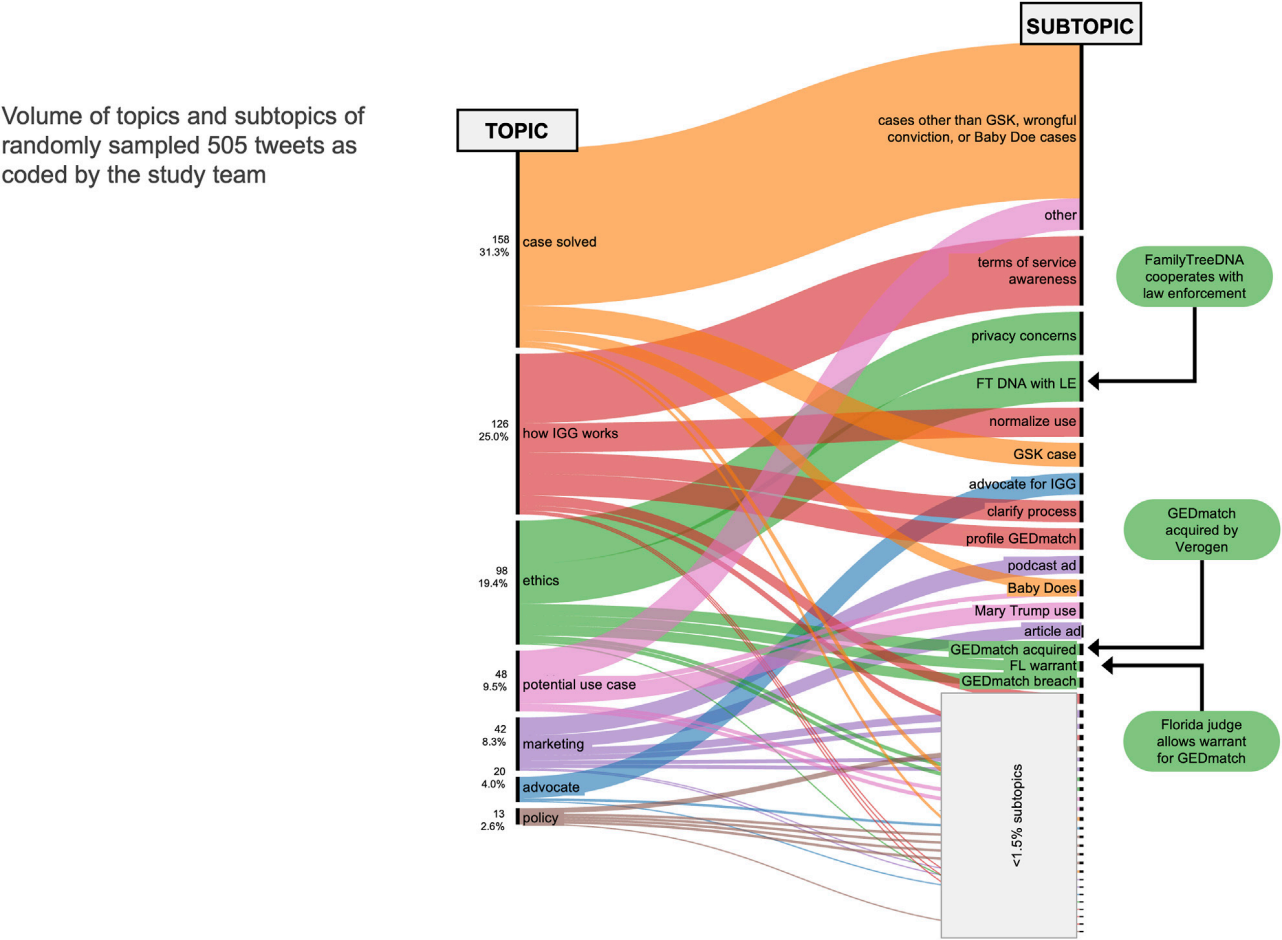
Proportion of topics and subtopics for randomly sampled tweets.

In addition to analysis of temporal conversations that spiked periodically over the 4.5 year period, we coded for the topics and sentiment of a range of conversations over time. The episodic events would be valuable for deep dives into a news event, but we also were interested in sentiment changes and shifts in topic over time. Coding of the 5% random sample set of 10,662 original posts and replies yielded 536 unique tweets. After topic coding, 31 coded irrelevant to IGG and were excluded, leaving 505 tweets for topic and sentiment coding. The remaining 505 tweets revealed seven overarching topics comprised of 42 subtopics, including the ten event subtopics comprising the top ten peak events (see Figure 4). Many tweets related to a particular case being solved (31.3%) or an open case that could potentially benefit from IGG (9.5%). Overall, 4.0% of the tweets were on the solving of the Golden State Killer case. Many of the tweets mentioned the Golden State Killer but were not solely focused on the resolution of the case; these were coded as one of the other subtopics. A quarter of the tweets (25.0%) explained how the IGG process works, including clarifications about terms of service of platforms used for genetic genealogy (10.9%) and details about key experts in IGG (5.0%). Nearly a fifth (19.4%) of the tweets were on ethics of IGG, with subtopics including privacy concerns (6.7%) and discussions on law enforcement access to FamilyTreeDNA (6.3%). The topics coded over time (see Supplementary Figure S2) show a steady stream of most topics including case resolutions and potential use cases, but seemingly more volume of discussion of the ethics of IGG at peak events and more on how IGG works in the early years of conversations.

### 3.3 Sentiment analysis

#### 3.3.1 Sentiment and IGG position analysis of random sample set

**FIGURE 5:**
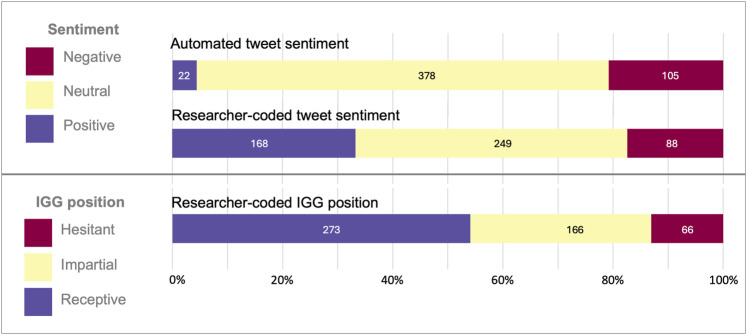
Proportion of randomly sampled tweets showing automated sentiment codes, researcher-coded sentiment, and IGG position.

In coding the random sample set, 12.9% (65/505) of the tweets needed resolution by the study team for sentiment and 7.1% (36/505) for IGG position. Manual coding of sentiment showed a 55.7% (271/505) concordance with automated sentiment codes. Our expert research coding was more likely to detect positive tone in tweets. Because of the discordance, our statistical analyses relied on the manually coded data, not on the automated sentiment codes. A positive sentiment code was applied by the study team to 33.3% of the random set of tweets; a neutral sentiment was applied to 49.3%, and a negative sentiment was applied to 17.4% of the tweets (see Figure 5). IGG position was noted as receptive for 54.1% of the tweets, impartial for 32.9%, and hesitant for 13.1%. The concordance pairs of positive-receptive, neutral-impartial, and negative-hesitant were 65.7% overall. A neutral code was applied to 23.4% of those tweets coding receptive/hesitant and an impartial code applied to 6.9% of those coding positive/negative. The remaining 4.0% were discordant as negative-receptive; no tweets coded positive-hesitant. We saw fluctuations in sentiment over time across the random sample set corresponding to the news cycles, but a gradual increase in the proportion of tweets receptive to IGG; given the fluctuation in sentiment and that only 5% of tweets were sampled, however, this finding is not necessarily wholly indicative of a trend (see Supplementary Figure S3).

#### 3.3.2 Sentiment and IGG position analysis of key events

**FIGURE 6:**
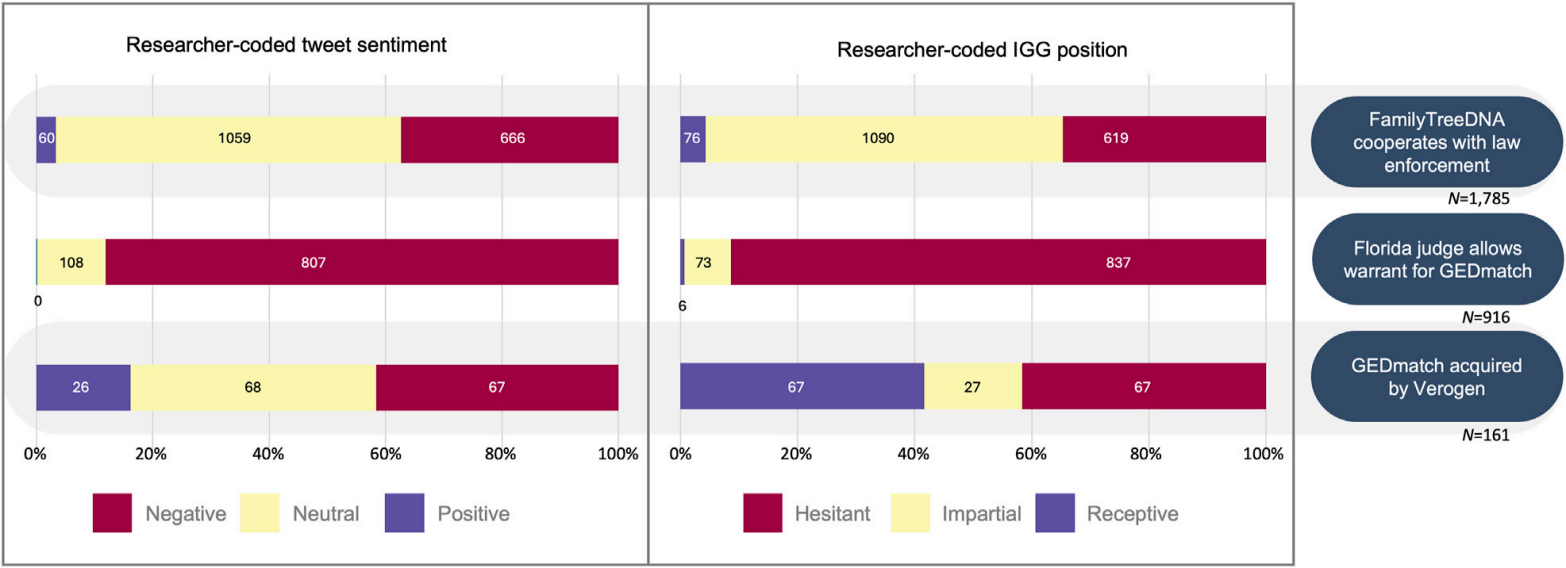
Proportion of tweets from three key events showing automated sentiment codes, researcher-coded sentiment, and IGG position.

We coded for sentiment and IGG position for the three datasets reflecting conversations on IGG processes that might be relevant to public opinion and policy. For the “FamilyTreeDNA cooperates with law enforcement” (FTDNA-LE) topic, we had 1,785 tweets; for the “Florida judge allows warrant for GEDmatch” (FL warrant) topic, we had 828 tweets; and for the “GEDmatch acquired by Verogen” (GEDmatch acquire) topic, we had 135 tweets (see Table 1). In coding these sample sets, 9.8% (35/355), 29.4% (15/51), and 22.7% (5/22) of the tweets needed resolution by the study team for sentiment, and 13.0% (46/355), 19.6% (10/51), and 13.6% (3/22) for IGG position, respectively. The sentiment and IGG position of these tweets skewed more negatively than the random sample set (see Figure 6).

**TABLE 1:** Select Twitter/X events analyzed.

Event	Filter terms	Tweets (posts, shares, replies)	Date range
FamilyTreeDNA cooperates with law enforcement	“familytree” OR “compan” OR (“family tree” AND “FBI”) OR “family-tree-dna”URL	1,785 (527, 1,223, 35)	01 Feb 2019 – 27 Mar 2019
Florida judge allows warrant for GEDmatch	“judge” OR “court” OR “granted” NOT “Verogen” NOT “owner”	828 (63, 759, 6)	05 Nov 2019 – 08 Nov 2019
GEDmatch acquired by Verogen	“Verogen” OR “profit” OR (“owner” NOT “warrant”)	135 (30, 98, 7)	10 Dec 2019 – 14 Jan 2020

### 3.4 Purpose of IGG tweets

We coded for purpose for all tweets that we coded for sentiment and include these codes in the dataset. We evaluated the trends in tweet purpose among the random sample set, finding a majority to be “share news” (274/505, 54.3%) and over a quarter being personal opinions (133/505, 26.3%) (see Supplementary Figure S4). We noted only 1 joke in the random dataset and no educational materials. Professional opinions comprised 6.9% (35/505) of the random set of tweets and marketing another 5.5% (28/505). The other codes for share experience, requests, and asking for advice were under 2.5% of the sample set.

## 4 Discussion

We authors and our expert colleagues learned of the DeAngelo arrest through media and social media; we also sought to correct our own understandings of the IGG process through news articles perpetuated *via* social media. So, we were not surprised to see that the tweets we identified on Twitter/X represented a range of conversations and perspectives.

We also were not surprised to see the peaks in conversations over time representing the events that we experts expected to see. The peak analyses were particularly invaluable to identify and characterize public discussion around prominent news events related to IGG over the 4.5 years since the explosion in public awareness after the arrest of the Golden State Killer. Some of the topics identified were known to the study team, for example, the acquisition of GEDmatch by Verogen and the news of the warrant to search GEDmatch. Other topics that we experts were aware of did not emerge in the peak analyses. For example, the publication of the 2018 Erlich study that demonstrated the comprehensiveness of IGG databases among Northern Europeans and the breach of GEDmatch in July 2020 did appear as subtopics in our random sampling but not in the peak analyses. We could surmise from this that either (a) these topics were of less interest to the public than the experts; (b) that these topics were diffuse across timeframes; or (c) that our search string did not sufficiently capture these topics. We can rule out (b) since the topics were detected in our random sampling, albeit with less volume than we would have expected (0.6% of tweets discussed the Erlich study, 1.6% of tweets were on the breach). We cannot rule out (c) that our search string was insufficient, but we feel confident in the scope of representation given the range of topics detected.

A large number (29.5%) of tweets were announcements of solved cases, with only 12.7% being tweets about solving the Golden State Killer case. The majority (78.5%) of the “case solved” tweets were on a range of over 70 cases. This is likely indicative of both the excitement and concern for the new tool as well as an attempt among IGG allies to inform the public of the utility of IGG. The 9.0% of tweets that focused on the potential use of IGG for select cold cases or applications could be a crowd-sourcing tool for selecting cases of public import.

Built into the Brandwatch platform is an automated sentiment analysis tool that codes tweets as positive, negative, or neutral based on a generalized language processing model. Since this model is meant for generalized text, it is expected that when looking at tweets which discuss especially negatively charged events, the model will more likely be incorrect (van Atteveldt et al., 2021). In the case of IGG we expected that the auto-generated sentiment would skew more negative than researcher-coded sentiment since the IGG tweets are often covering horrific crimes and deaths. Indeed, we noted more negative-coded tweets by the software (20.8%) than the human researchers (17.4%); however, we were surprised to see how few tweets were coded positive by the software (4.4%) versus the humans (33.3%). This is consistent with the hypothesis that the language model skews towards negative sentiment when discussing negative topics. Future methodology using automated sentiment measures should consider if the subject of discussion will skew results. More specialized models might be necessary to research opinion, like our use of “IGG position” presented here.

Among the random sample set of tweets, the greatest number of tweets was observed to have the purpose to “share news”, which is not surprising since it is known that many individuals utilize social media to learn about the news (Walker and Eva Matsa, 2021). That said, the number of tweets sharing news was twice that of the next largest category, implying that in this context many individuals are newly learning about the topic, or are aiming to further share news as practices shift. Among the tweets sharing news, it appeared that a majority (54.3%) were receptive to IGG. The nature of this correlation and its cause is unclear. It is possible that news headlines are mostly sharing positive news cases where IGG was used to solve a crime, or it could be that the type of individuals who are following the news on this emergent technology might be more receptive to its use overall.

The second largest purpose code was “personal opinion”, comprising 26.3% of the random sample set. For these tweets, it would be expected that there is reasonable diversity of opinions on Twitter/X. Overall, it was found that personal opinions did vary greatly in their IGG position, with a general skew towards those who were receptive to IGG. Notably though, is that the IGG positions within the personal opinion code were shown to be more mixed than in the share news category. This implies that individuals who are simply stating opinions regarding IGG without intending on sharing news are more likely to be hesitant regarding IGG than those who are actively sharing news of the technology. The overall opinions still appear to be receptive to IGG though, despite there being more individuals who are hesitant.

The tweets focusing on “how IGG works” and the “ethics of IGG” are a rich source of commentary to understand public viewpoints, both of experts and nonexpert citizens. Over time these subtopics cluster in an interesting pattern, with seemingly more tweets in the early years of IGG and potentially in alignment with key events. This could be studied further to determine whether the ethics concerns have been allayed over time. We do see an incline in both positive sentiment and receptive position towards IGG (Supplementary Figure S3), indicating increasing support for IGG. This incline is particularly notable for receptive position towards IGG, as reflected in the overall receptive support among 54.1% of the tweets. Indeed, with this study, we found the automated sentiment codes to be less useful than the researcher-coded sentiment analyses for text tone and for IGG position.

Despite the slight majority support for IGG, support seems heavily dependent on the topic of discussion, as shown by our analyses of three key events for policy interest (Figure 6). Both the researcher-coded sentiment and IGG position skewed negative (88.2%) and hesitant (91.4%), respectively, for the 916 tweets collected on the subtopic “Florida judge allows warrant of GEDmatch.”

A deeper dive into the perspectives on the “ethics of IGG” and “how IGG works” might inform policy directives, as would analyses of the misinformation spread within these realms and beyond. During tweet coding, we noted anecdotally that many individuals appeared to be misinformed regarding the use of IGG or how it works, stemming our next phase of research into misinformation within these data, now underway. Further research on what these misconceptions may be or how these misconceptions may be related to position on IGG could yield some interesting results. Unfortunately, direct conversation about policy developments was lacking with only 2.6% of the randomly selected tweets discussing state legislation for IGG and federal policy actions.

We cannot be certain that our search string captured the entirety of tweets on IGG, especially since social media is fluid with tweets potentially being deleted by the user. This is particularly relevant in examining the trends in conversations over time. Importantly, since our study was examining an emerging technology, we most likely failed to capture tweets written in the early weeks and months before a common terminology was established. Even among practitioners and experts, it has taken many years for a common term for the IGG approach to crystalize, with options ranging from “forensic investigative genetic genealogy” to “long range familial searches”, among others (Tuazon et al., 2024).

The amount of data generated from our data pull could not be qualitatively characterized in its entirety, which is why we opted to randomly select tweets across the timeline to give an overview of the findings. Many avenues for future research remain. In fact, since Twitter was purchased and rebranded as X directly after our 2022 data pull, the data we collected and make available with this publication might no longer be accessible online. Any future research might require a current update to the dataset utilizing a similar search string, but since many individuals have left Twitter/X and many tweets have been lost over time, Twitter/X might no longer prove to be as random or representative of a sample as the original dataset we present here.

The analysis we conducted primarily was aimed at understanding the content of people’s tweets in a bubble, generally understanding each tweet as a stand-in for an individual’s thought. That said, tweets do not exist in a vacuum; they exist online where they are circulated and shared. Some tweets get shared more than others, and many tweets are coopted by discrete communities. Further research can utilize social network analysis to understand who is talking about IGG online and what communities appear to share tweets on the topic more. The impact of tweets could also be analyzed to understand if certain opinions get shared or read more than others. For example, it could be possible that there are more individuals making unique tweets receptive to IGG but that the tweets hesitant to IGG get more shares or more likes. Moreover, many individuals read through social media all the time without ever posting themselves.

This dataset of Twitter/X tweets over the first 4.5 years of the use of IGG represents contains a trove of qualitative data that we make available for further research, redacted to an extent for privacy purposes (Williams et al., 2017). We characterized the scope of the data by categorizing topics discussed within Twitter/X, particular events that sparked peaks in social media discussion, and the general sentiment or tone of the conversations. We created for this dataset a separate qualitative measure of “IGG Position” to gauge the general receptiveness towards IGG as a law enforcement tool among Twitter users. We make the full dataset available for further analyses at the request of other researchers. The replicability might be a challenge given that Twitter was rebranded X days after our data pull and many tweets and users have now been removed from the platform. These data could provide insight are insightful for this particular topic of IGG because of the rapidly shifting policy that has developed in reaction to the public’s fears of law enforcement overreach and concerns that private consumer genomics platforms are being accessed by law enforcement without permission of the users. Some of the fears have been found to be based in misconceptions, while others might be founded in truth or partial truths. This dataset will be invaluable to further pursuit to untangle the gaps in understanding of how IGG works. While these data were developed for our research team to evaluate a topic of interest, the strategy used is applicable to other topics of interest, particularly for emerging tools, technologies, ideas, or concepts.

## Data Availability

The datasets presented in this study can be found in online repositories. The names of the repository/repositories and accession number(s) can be found in the article/Supplementary Material.
