# Supplementary material for: Insights from social media into public perspectives on investigative genetic genealogy
Source: Front Genet. 2025 Jan 6;15:1482831. doi: 10.3389/fgene.2024.1482831 (PMC11743634; doi:10.3389/fgene.2024.1482831)
Supplement: Supplementary file 1 [file Table2.docx]

Supplementary Material

**1 Supplementary Data**

The datasets generated for this study can be found in the ForenSeq Study (R01HG011268) OSF repository (<https://osf.io/n9tfu/>). Raw data containing identifiers (e.g., Twitter/X user names) are not available for public review (Williams, Burnap, and Sloan 2017). Posts are archived with usernames removed to prevent identification. All data are available upon request to the corresponding author; data will be shared in keeping with appropriate human subjects protections.

**2 Supplementary Figures and Tables**

**Supplementary Table S1**. Brainstormed terms for Twitter/X advanced searches

| **General** | **Law enforcement-related** | **DNA-related** | **Genealogy-related** |
| --- | --- | --- | --- |
| investigative genetic genealogy | law enforcement | DNA | genetic genealogy* |
| investigative genealogy | ~~LE~~ | genetic* | genealogy* |
| forensic genetic genealogy | cop | ~~genetic database~~ | family tree |
| forensic genealogy | cops |  | relative* |
| IGG | detective* |  | ancestr* |
| FGG | police |  | familial DNA |
|  | criminal* |  | familial searching |
|  | ~~perp~~ |  | GEDmatch |
|  | perpetrator* |  | Family Tree DNA |
|  | suspect* |  |  |
|  | victim* |  |  |
|  | cold case* |  |  |
|  | crime* |  |  |
|  | investigation* |  |  |
|  | ~~solve~~ |  |  |
|  | forensic* |  |  |
| Strikethrough terms were brainstormed but not searched. Common misspellings of company names were considered. | | | |

**Supplementary Table S2**. Advanced search results

| **Included** | **General** | **Law enforcement-related** | **DNA-related** | **Genealogy-related** | **Age of 10^th^ tweet** | **Number of relevant tweets** |
| --- | --- | --- | --- | --- | --- | --- |
| ✔ | investigative genetic genealogy |  |  |  | 10 days | 100% |
| ✔ | investigative genealogy |  |  |  | 1.5 months | 100% |
| ✔ | forensic genetic genealogy |  |  |  | 13 days | 100% |
| ✔ | forensic genealogy |  |  |  | 5 days | 100% |
|  | IGG |  |  |  | 42 minutes | none |
|  | FGG |  |  |  | 6 hours | none |
|  |  | law enforcement |  | family tree | 8 months | 50% |
| ✔ |  | law enforcement |  | genetic genealogy | 27 days | 100% |
|  |  | cop |  | family tree | 24 days | 20% |
| ✔ |  | cop | DNA | family tree | 2.5 years | 100% |
|  |  | cop | genetic | family tree | 3.5 years | 70% |
| ✔ |  | cop |  | genetic genealogy | 4 months | 100% |
|  |  | cops |  | family tree | 24 days | 10% |
| ✔ |  | cops |  | genetic genealogy | 1 year | 90% |
|  |  | detective |  | family tree | 26 days | none |
| ✔ |  | detective |  | genetic genealogy | 1.5 month | 100% |
|  |  | police |  | family tree | 16 days | 10% |
| ✔ |  | police | DNA | family tree | 5 months | 70% |
| ✔ |  | police | genetic | family tree | 2.5 years | 100% |
| ✔ |  | police |  | genetic genealogy | 6 days | 100% |
|  |  | criminal |  | family tree | 19 days | none |
| ✔ |  | criminal |  | genetic genealogy | 18 days | 100% |
|  |  | perpetrator |  | family tree | > 2 years | 50% |
| ✔ |  | perpetrator |  | genetic genealogy | 5 months | 100% |
|  |  | suspect |  | family tree | 12 days | none |
| ✔ |  | suspect |  | genetic genealogy | 24 days | 100% |
|  |  | victim |  | family tree | 1 month | none |
| ✔ |  | victim |  | genetic genealogy | 3 months | 100% |
| ✔ |  | cold case |  | family tree | 6 months | 100% |
| ✔ |  | cold case |  | genetic genealogy | 14 days | 100% |
|  |  | crime |  | family tree | 8 days | none |
| ✔ |  | crime |  | genetic genealogy | 15 days | 100% |
|  |  | investigation |  | family tree | 1.5 months | 10% |
|  |  | investigation | DNA | family tree | 1.5 years | 40% |
|  |  | investigation | genetic | family tree | 3.5 years | 60% |
| ✔ |  | investigation |  | genetic genealogy | 3 months | 100% |
| ✔ |  |  |  | familial DNA | 5 days | 80% |
|  |  |  |  | familial searching | 6 months | 20% |
| ✔ |  | law enforcement |  | ancestry | 1 month | 100% |
|  |  | cop |  | ancestry | 17 days | 20% |
|  |  | detective |  | ancestry | 12 days | none |
|  |  | police |  | ancestry | 2 days | 70% |
|  |  | criminal |  | ancestry | 5 days | none |
|  |  | perpetrator |  | ancestry | 5 months | none |
|  |  | suspect |  | ancestry | 8 days | 20% |
|  |  | victim |  | ancestry | 3 days | 40% |
| ✔ |  | cold case |  | ancestry | 7 weeks | 100% |
|  |  | crime |  | ancestry | 19 hours | 40% |
|  |  | investigation |  | ancestry | 2 weeks | 10% |
| ✔ |  | law enforcement |  | GEDmatch | 3 months | 100% |
| ✔ |  | cop |  | GEDmatch | 1.5 years | 100% |
| ✔ |  | cops |  | GEDmatch | 1.5 years | 100% |
| ✔ |  | detective |  | GEDmatch | 11 months | 70% |
| ✔ |  | police |  | GEDmatch | 5 months | 100% |
| ✔ |  | criminal |  | GEDmatch | 1.5 years | 100% |
| ✔ |  | perpetrator |  | GEDmatch | > 4 years | 100% |
| ✔ |  | suspect |  | GEDmatch | 6 months | 90% |
| ✔ |  | victim |  | GEDmatch | > 2 years | 100% |
| ✔ |  | cold case |  | GEDmatch | 13 months | 100% |
| ✔ |  | crime |  | GEDmatch | 4 months | 100% |
| ✔ |  | investigation |  | GEDmatch | > 2 years | 100% |
| ✔ |  | law enforcement |  | Family Tree DNA | 2 years | 100% |
| ✔ |  | cop |  | Family Tree DNA | > 4 years | 50% |
| ✔ |  | cops |  | Family Tree DNA | > 4 years | 50% |
| ✔ |  | detective |  | Family Tree DNA | > 4 years | 100% |
| ✔ |  | police |  | Family Tree DNA | > 2 years | 100% |
| ✔ |  | criminal |  | Family Tree DNA | > 3 years | 100% |
|  |  | perpetrator |  | Family Tree DNA | no results |  |
| ✔ |  | suspect |  | Family Tree DNA | > 3 years | 90% |
| ✔ |  | victim |  | Family Tree DNA | > 3 years | 30% |
|  |  | cold case |  | "Family Tree DNA | no results |  |
| ✔ |  | crime |  | Family Tree DNA | > 2 years | 100% |
| ✔ |  | investigation |  | Family Tree DNA | > 4 years | 60% |
|  | All searches conducted 8/29/22 to 9/15/22 |  |  |  |  |  |

**Supplementary Table S3**. Twitter search results

| **Tweets** | **Gender** | **Thread type** |
| --- | --- | --- |
| 24,209 | Male 5,824  Female 6,470  Unknown 11,915 | Post 7,816  Share 13,490  Reply 2,903 |

**Supplementary Table S4**. Twitter bins

| **Bin number** | **Date range** | **Tweets (posts, shares, replies)** | **Top 25 peaks*** |
| --- | --- | --- | --- |
| 1 | Apr 01 2018 – Aug 19 2018 | 3,906 (1,325, 2,057, 524) | 1, 10, 13, 16 |
| 2 | Aug 20 2018 – Dec 10 2018 | 1,853 (612, 1,152, 89) | 3, 19, 22 |
| 3 | Dec 11 2018 – Mar 31 2019 | 3,616 (1,046, 2,343, 227) | 2, 7, 8 |
| 4 | Apr 01 2019 – Aug 19 2019 | 2,350 (795, 1,279, 276) | 15, 18, 25 |
| 5 | Aug 20 2019 – Mar 30 2020 | 4,197 (1,287, 2,545, 365) | 5, 9, 11, 23 |
| 6 | Mar 31 2020 – Feb 07 2021 | 2,705 (942, 1,217, 546) | 14, 17, 24 |
| 7 | Feb 08 2021 – Aug 19 2021 | 1,485 (640, 649, 196) | 21 |
| 8 | Aug 20 2021 – Jan 12 2022 | 1,141 (291, 661, 189) | 6, 12 |
| 9 | Jan 13 2022 – Oct 25 2022 | 2,843 (866, 1,497, 480) | 4, 20 |
| *Peaks were numbered from highest volume (1) to lowest volume (25) | | | |

**Supplementary Table S5**. Autoclusters within each bin

| **Bin number** | **Specific clusters** | **Nonspecific cluster** |
| --- | --- | --- |
| 1 | How IGG works: familial DNA use – 131  Case solved: Golden State Killer arrest – 772  Advocate for IGG: expand IGG – 63  Case solved: Golden State Killer arrest – 92  Potential use case: Delphi killer – 33  Case solved: Tinsley case – 58 | 2,757 |
| 2 | Ethics of IGG: blog – 28  Case solved: NorCal rapist – 148  Case solved: Fleming arrest – 63  How IGG works: Erlich paper – 82  Potential use case: adoptees – 57  How IGG works: GEDmatch feature – 195  Marketing: podcast – 91  Case solved: Taylor arrest – 148  Case solved: Tinsley case – 35 | 1,147 |
| 3 | Advocate for IGG: Kamala Harris article – 164  Ethics of IGG: FTDNA cooperates with LE – 791  How IGG works: GEDmatch feature article – 99 | 2,562 |
| 4 | Advocate for IGG: encourage expansion – 80  Case solved: Seattle cold case – 56  Ethics of IGG: concerns about consent – 97 | 2,117 |
| 5 | Policy: DOJ policy – 65  Ethics of IGG: Florida judge allows warrant of GEDmatch – 686  How IGG works: Erlich paper – 84  Potential use case: neonaticides – 73  Ethics of IGG: GEDmatch acquired by Verogen – 111  Advocate for IGG: gift DNA kits – 42  Case solved: Idaho case – 44 | 3,026 |
| 6 | Ethics of IGG: GEDmatch data leak – 86 | 2,619 |
| 7 | -- *no clusters emerged* -- | 1,485 |
| 8 | Advocate for IGG: clear sexual assault kits – 117  Case solved: wrongful conviction – 86 | 825 |
| 9 | Case solved: Pennsylvania cold case – 243  How IGG works: profile of Schubert – 220  Case solved: Fort Bend case – 36 | 2,344 |

**Supplementary Table S6**. Highest 25 peaks

| **Number** | **Peak event** | **Approx. date** | **Approx. # tweets** | **Bin** |
| --- | --- | --- | --- | --- |
| 1 | Case solved: Golden State Killer arrest | Apr 25 2018 | 950 | 1 |
| 2 | Ethics of IGG: FTDNA cooperates with LE | Feb 01 2019 | 760 | 3 |
| 3 | How IGG works: GEDmatch feature article | Oct 15 2018 | 430 | 2 |
| 4 | Case solved: Pennsylvania cold case | May 30 2022 | 410 | 9 |
| 5 | Ethics of IGG: Florida judge allows warrant of GEDmatch | Nov 05 2019 | 340 | 5 |
| 6 | Case solved: wrongful conviction | Oct 22 2021 | 220 | 8 |
| 7 | Advocate for IGG: Kamala Harris article | Jan 15 2019 | 160 | 3 |
| 8 | Ethics of IGG: FTDNA cooperates with LE | Feb 25 2019 | 140 | 3 |
| 9 | Ethics of IGG: GEDmatch acquired by Verogen | Dec 10 2019 | 140 | 5 |
| 10 | Case solved: Gold State Killer arrest | May 19 2018 | 135 | 1 |
| 11 | Ethics of IGG: GEDmatch acquired by Verogen | Dec 17 2019 | 125 | 5 |
| 12 | Potential use case: clear sexual assaults | Oct 07 2021 | 110 | 8 |
| 13 | Potential use case: Delphi killer | Jun 01 2018 | 105 | 1 |
| 14 | Ethics of IGG: GEDmatch data leak | Jul 19 2020 | 105 | 6 |
| 15 | Case solved: Seattle cold case | May 07 2019 | 100 | 4 |
| 16 | How IGG works: use of ancestry sites | Jun 28 2018 | 100 | 1 |
| 17 | Potential use case: Mary Trump paternity case | Sep 12 2020 | 95 | 6 |
| 18 | Ethics of IGG: concerns for consent | May 16 2019 | 90 | 4 |
| 19 | Case solved: Fleming arrest | Sep 23 2018 | 90 | 2 |
| 20 | Case solved: Days Inn murder | Apr 05 2022 | 90 | 9 |
| 21 | Case solved: Montana cold case | Jun 10 2021 | 85 | 7 |
| 22 | Marketing: Podcast on IGG | Nov 05 2018 | 80 | 2 |
| 23 | Policy: DOJ rules established | Sep 25 2019 | 75 | 5 |
| 24 | Ethics of IGG: GEDmatch data leak | Jul 22 2020 | 75 | 6 |
| 25 | Advocate for IGG: 1-yr since GSK arrest | May 21 2019 | 70 | 4 |

**Supplementary Table S7**. Topics and subtopics coded from random sample set (N=505)

| **Topic** | **Subtopic** | **Tweets** |
| --- | --- | --- |
| advertisement | article | 10 |
| advertisement | blog | 3 |
| advertisement | book | 3 |
| advertisement | conference | 6 |
| advertisement | IGG company | 4 |
| advertisement | podcast | 14 |
| advertisement | suggest podcast | 1 |
| advertisement | TV | 1 |
| advocate for IGG | advocate generally | 17 |
| advocate for IGG | job desire | 2 |
| advocate for IGG | willingness to participate | 1 |
| ethics of IGG | concern for privacy | 34 |
| ethics of IGG | concern for racism | 3 |
| ethics of IGG | Erlich study | 3 |
| ethics of IGG | FL warrant | 8 |
| ethics of IGG | PA warrant | 1 |
| ethics of IGG | breach of GEDmatch | 8 |
| ethics of IGG | GEDmatch purchase | 9 |
| ethics of IGG | FT DNA work with LE | 32 |
| how IGG works | terms of service awareness | 55 |
| how IGG works | clarify process | 17 |
| how IGG works | normalize use | 23 |
| how IGG works | question process | 4 |
| how IGG works | profile IGG people | 7 |
| how IGG works | profile IGG company | 18 |
| how IGG works | training in IGG | 1 |
| how IGG works | success metrics | 1 |
| policy | Maryland | 4 |
| policy | Montana | 1 |
| policy | New York | 2 |
| policy | California | 2 |
| policy | DOJ | 2 |
| policy | Kamala Harris | 2 |
| potential use case | Baby Doe | 5 |
| potential use case | Mary Trump | 13 |
| potential use case | unidentified | 7 |
| potential use case | other | 23 |
| case solved | Golden State Killer | 20 |
| case solved | Baby Doe | 9 |
| case solved | wrongful conviction overturned | 3 |
| case solved | genetic witness experience | 2 |
| case solved | other | 124 |

|  |
| --- |

**Supplementary Figure S1.** Temporal bins used to break up the data for peak analyses.

|  |
| --- |

**Supplementary Figure S2.** Temporal volume of topics of the random sample set of 505 relevant tweets.

|  |
| --- |

**Supplementary Figure S3.** Temporal steam graph showing the proportion of tweets coded for sentiment and IGG position of random sample set of 505 relevant tweets.

|  |
| --- |

**Supplementary Figure S4.** Temporal volume of purpose codes of the 505 randomly sampled tweets.
