# Supplementary material for: Insights from social media into public perspectives on investigative genetic genealogy
Source: Front Genet. 2025 Jan 6;15:1482831. doi: 10.3389/fgene.2024.1482831 (PMC11743634; doi:10.3389/fgene.2024.1482831)
Supplement: Supplementary file 2 [file DataSheet1.pdf]

Qualitative Codebook for Tweets on Investigative Genetic Genealogy

Process

- Two to three coders independently review a batch of tweets and code according to the codebook
- Coders flag any posts that are particularly challenging to code (discuss) in the applicable column
- Coders flag for triggering but relevant material (coder may choose to disengage from these tweets and flag for later discussion)
- Coders compare to identify agreement/disagreement on codes, discuss, refine codebook, and assign final codes

General Guidelines

- Codes are based on the content of the tweet itself. Descriptions of codes below thus reference the tweet and not the Twitter user.
- Review of contextualizing content such as attachments, threads, or replies may be used as supporting evidence for code assignment, but the final determinant of the code is the content of the tweet as it would appear in a Twitter feed.

Topic Considerations

- This codebook was refined for Twitter analyses of a dataset of tweets collected between April 2018 and October 2022 on the use of Investigative Genetic Genealogy to investigate crimes and resolve identities of human remains.
- Traditional sentiment codes were used to code the text for tone. A separate sentiment code of IGG Position was developed to code for the tweet's perceived receptivity to the practice of IGG.

**Post purpose codes**	Weighs both the probable intentions of the Twitter user and the probable reception of the tweet by a general audience. Probable reception takes precedence.
Share experience	• Tweet shares a personal or a second-hand experience related to an aspect of IGG
Personal opinion	• Tweet shares a personal opinion about an aspect of IGG<br>• Tweet may explicitly claim the opinion (e.g., "I think," "I believe")<br>• Tweet may not explicitly claim an opinion, but the content is interpreted as an implicit endorsement (e.g., "Here's a smart article about …")<br>• Tweet may be a definitive statement, but it also may have a musing tone without being an explicit request for information or advice (e.g., "Reading this article makes me wonder …")<br>• The Twitter user's account might be either a personal account or a professional account, where a general audience would not understand the professional to be able to claim authority (e.g., a tech journalist)<br>• May include sarcasm, humor, or irony ***[See FLAG code below]***
Professional opinion	• A professional Twitter user in a relevant position of authority shares a statement about an aspect of IGG<br>• The Twitter user's authority might be scientific or otherwise (e.g., a member of law enforcement, a genetic genealogist, an ethicist). 'Authority' means that members of the public are likely to give weight to the account's statements about IGG<br>• Claims to authority by the Twitter user may be included in the username, text, attachments, or bio (e.g., use of a title like "geneticist;" references to organizations or

	institutions with authority like universities, references to professional events like conferences, etc.)<br>• Exclude quotations from professionals pulled from newspaper articles
Ask opinion	• Tweet asks the general audience or a specific group for information related to IGG<br>• Tweet may request general or personalized information<br>• Tweet may ask for the perspective or opinion of other users
Request	• "you need to…" or "can you help me do …"<br>• Tweet makes a call for action that requires the support of a broad public or a directed tweet calling for action (e.g., "Use IGG to solve this case")<br>• The call for action requires support beyond the user's ability, excluding actions that individuals may take on their own<br>• Requests may be directed at a particular person or group through the text, directed mentions, replies, etc.<br>• Excludes requests to share or retweet, as this is part of the implied purpose of social media and when explicit, falls under share news
Share news	• Tweet shares news or a recent announcement related to IGG (e.g., cases solved, new applications, company or government policy)<br>• News includes newly discovered information to be disseminated<br>• News may include attachments in the form of a link to an article or video from a news outlet or a screenshot of a social media message or post (e.g., a What'sApp message, another tweet)<br>• Not all tweets with attachments, even attachments of news articles, have news sharing as their main purpose. The tweet content is the key determinant of whether a tweet is coded as "share news"<br>• Not all "share news" tweets include attachments
Educational material	• Tweet shares an academic article related to an aspect of IGG
Marketing	• Tweet that shares content that promotes a product or service related to an aspect of IGG (e.g., endorsement of a company that provides IGG services)<br>• Note that promotions were excluded in our search filters, but some posts may still fit these criteria
Joke	• Tweet makes a joke or makes a humorous or ironic comment related to an aspect of IGG
Excluded	• Tweets that defy coder's ability to assign an accurate code due to comprehension challenges (e.g., tweet includes slang, dialect, or non-standard spelling that cannot be interpreted)<br>• Non-English tweets that may have been captured despite the English-language only search filter<br>• Spam, e.g., pornography

**Position on IGG codes**	Describes receptiveness with reference to IGG as a tool for law enforcement use, while sentiment refers to the entire tweet with context.
Receptive	• Tweet is in support of the use of IGG<br>• Support might be expressed for IGG overall, for its use in a specific context, or aspects of the process
Hesitant	• Tweet expresses doubts or concerns around an aspect of IGG<br>• Doubts and concerns might range in strength from mild concern to opposition<br>• Excludes tweets asking for information to clarify science or process
Impartial	• Tweet does not contain a message or phrasing that could be interpreted as an attitude towards IGG<br>• Tweet might contain language that could easily be interpreted as either advocating for or questioning the use of IGG

Sentiment codes	Describes the overall tone of the tweet. The difference between position and sentiment is that position is always with reference to IGG, while sentiment refers to the entire tweet with context.
Positive	• Tweet includes words, phrases, or punctuation that taken together indicate an overall positive tone
Negative	• Tweet includes words, phrases, or punctuation that taken together indicate an overall negative tone
Neutral	• Neither a positive nor negative tone is indicated

**Flag**	
Discuss	• Flag for discussion between coders; used for any particularly challenging tweets
Sarcasm	• Flag for tweets with a sarcastic tone
Triggering	• Flag for tweets that are relevant but contain emotionally challenging content for the coder
